# Robust Achromatic All-Dielectric Metalens for Infrared Detection in Intelligent Inspection

**DOI:** 10.3390/s22176590

**Published:** 2022-08-31

**Authors:** Wenrong Si, Zhengyong Hu, Dun Lan, Yi Zhou, Wei Li

**Affiliations:** 1 State Grid Shanghai Electrical Power Research Institute, Shanghai 200437, China; 2 State Key Laboratory of Functional Materials for Informatics, Shanghai Institute of Microsystem and Information Technology, Chinese Academy of Sciences, Shanghai 200050, China; 3 University of Chinese Academy of Sciences, Beijing 100049, China

**Keywords:** metalens, achromatic, infrared detection

## Abstract

Metalens has the advantages of high design freedom, light weight and easy integration, thus provides a powerful platform for infrared detection. Here, we numerically demonstrated a broadband achromatic infrared all-dielectric metalens over a continuous 800 nm bandwidth, with strong environmental adaptability in air, water and oil. By building a database with multiple 2π phase coverage and anomalous dispersions, optimizing the corrected required phase profiles and designing the sizes and spatial distributions of silicon nanopillars, we numerically realized the design of broadband achromatic metalens. The simulation results of the designed metalens show nearly constant focal lengths and diffraction-limited focal spots over the continuous range of wavelengths from 4.0 to 4.8 μm, indicating the ability of the designed metalens to detect thermal signals over a temperature range from various fault points. Further simulation results show that the metalens maintains good focusing performance under the environment of water or oil. This work may facilitate the application of metalens in ultra-compact infrared detectors for power grid faults detection.

## 1. Introduction

Electric energy is an indispensable part of national production and life. In order to ensure the power supply quality of the power grid, it is necessary to detect faults in the power grid in time. Using optical method to detect power grid faults not only meets the requirements of detection without power cut, but also has the advantage of easy determination of the fault points. Thermal signals from fault points can be detected by means of infrared detection. However, the traditional optical systems of infrared detection are bulky and heavy, which is not convenient for practical applications, especially for the intelligent inspection with drones.

The development of metasurfaces provides a new paradigm for optical wavefront control. A metasurface is an artificially designed functional device with subwavelength structures arranged in a specific way on a two-dimensional plane [1]. The amplitude, phase and polarization of the incident wave can be controlled by adjusting the size and spatial distribution of the subwavelength structures. Metasurfaces have the advantages of excellent interfaces-control ability, high design freedom, small thickness, light weight, easy integration and simple fabrication. Purposedly designed metasurface functioning as a lens is named as metalens, which realizes light focusing and imaging [2,3,4,5,6,7,8,9,10,11,12,13,14,15,16,17,18,19,20,21,22,23,24,25,26,27,28,29,30,31,32,33]. Benefit from the advantages of metasurface, metalens is suitable for infrared detection. Thanks to its planar configuration, metalens is also suitable for integration with CMOS image sensor to realize ultra-compact optical system. To be further utilized in practical applications, the operating band of metalens should be enlarged to detect thermal signals over a temperature range from various fault points.

Several achromatic metalenses have been studied [8,16,17,18,19,20,21,22,23,24,25,30,31,32,33]. In 2015, Federico Capasso et al. proposed a multiwavelength achromatic metalens at wavelengths of 1330, 1550 and 1800 nm by dispersive phase compensation, based on low-loss dielectric resonators, [8]. In 2017, Xiangang Luo et al. realized a multiwavelength achromatic metalens working at visible wavelengths of 473 nm, 532 nm and 632.8 nm [17]. In the same year, Federico Capasso et al. proposed a narrow-band achromatic reflective metalens realizing a continuous 60 nm bandwidth in the visible, using titanium dioxide nanopillar (with a square cross-section) on a metallic mirror with a thin layer of silicon dioxide in-between [16]. In 2018, Nanfang Yu et al. developed a design methodology and created libraries of meta-units—building blocks of metasurfaces—with complex cross-sectional geometries to provide diverse phase dispersions (phase as a function of wavelength), creating broadband achromatic metalens, with operating band from 1200 nm to 1650 nm [19]. In the same year, Federico Capasso et al. demonstrated diffraction-limited achromatic focusing and achromatic imaging from 470 to 670 nm [18], while almost at the same time Din Ping Tsai et al. demonstrated complete elimination of chromatic aberration from 400 to 660 nm [20]. In 2021, Shumin Xiao et al. reported a broadband achromatic metalens with operating band from 650 nm to 1000 nm for near-infrared biological imaging window [32].

Most of the previous reports in recent years have focused on visible and near-infrared regions. However, mid-infrared broadband achromatic metalenses are also worth studying due to their potential applications in intelligent inspection. In this work, we numerically demonstrated a broadband achromatic infrared all-dielectric metalens over a continuous 800 nm bandwidth, with environmental adaptability in air, water and oil. Here, particle swarm optimization algorithm is employed to optimize the phase profile of achromatic metalens and the sizes and spatial distribution of thousands of silicon nanoposts. Then, three-dimensional finite-difference time-domain (FDTD) method is employed to simulate the optical characteristics of the metalens, showing near-diffraction-limit focal spots for normally incident light at the same focal plane over a continuous range of wavelengths from 4.0 to 4.8 μm. The broad bandwidth makes the metalens able to detect thermal signals over a temperature range from various fault points. To give a general view of the environmental adaptability of the proposed metalens, the background material is changed from air to water and oil, and the simulation results show that the metalens maintains good focusing performance under these environments. The cases of metalenses serving as elements of sensors or measurement systems are demonstrated in previous reports [34], including depth sensor using spatially multiplexed micro-metalens array based on light field imaging principles by Din Ping Tsai et al. [24], compact single-shot metalens three-dimensional (3D) depth sensor inspired by the eyes of jumping spiders using defocused images to reconstruct depth information by Federico Capasso et al. [35], single-shot quantitative phase gradient microscopy with phase gradient sensitivity better than 92.3 mrad μm^−1^ using a double-sided flat optic system with three optimized metalenses by Andrei Faraon et al. [36], dielectric metalens realizing wide-field depth retrieval with 3D imaging and distance measurements based on specially engineered point spread function (PSF) by Frank Setzpfandt et al. [37], Hartmann-Shack wavefront sensor using arrays of silicon-based metalenses realizing phase-gradient profiles detection by Jinsong Xia et al. [38], and compact spectrometer composed of three reflective dielectric metalenses resolving more than 80 spectral points from 760 to 860 nm by Andrei Faraon et al. [39]. This work may facilitate the application of metalens in ultra-compact infrared detectors for power grid faults detection.

## 2. Methods

**Figure 1:**
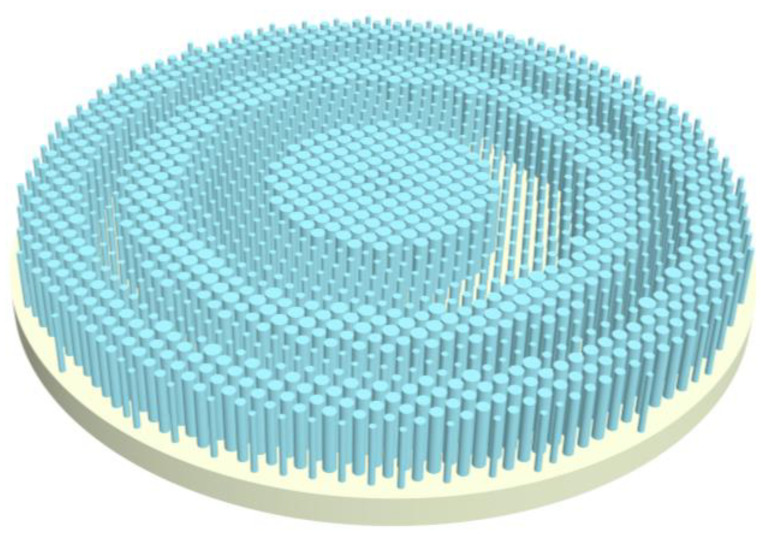
Schematic structure of the metalens.

**Figure 2:**
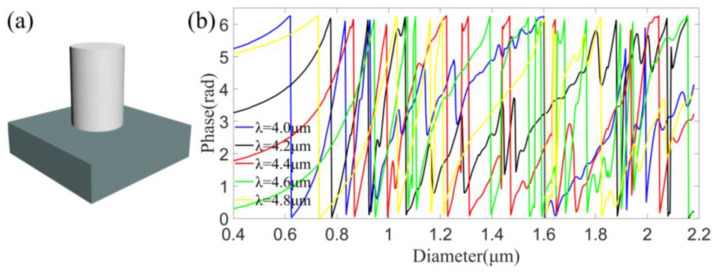
(**a**) Schematic of the unit cell, composed of a silicon nanopillar on fused silica substrate. (**b**) Simulated phase shifts (folded between 0 and 2π) as functions of the nanopillar diameters at wavelengths of 4.0 μm (blue), 4.2 μm (black), 4.4 μm (red), 4.6 μm (green) and 4.8 μm (yellow), i.e., the database.

As mentioned above, metalens is a two-dimensional array composed of optical scattering structures with subwavelength feature sizes. The main function of a metalens is to control the wavefront of light according to the phase profile induced by the spatial distributions of scattering structures of various sizes. The schematic structure of the metalens is shown in Figure 1. The unit cell of the metalens, i.e., the optical scattering structure, is a silicon nanopillar on fused silica substrate, shown in Figure 2a. The height of the silicon nanopillar is fixed as 8.7 μm, and the diameters of the nanopillars vary from 0.4 to 2.2 μm, and the size of unit cell is 2.4 μm. The refractive indexes of silicon nanopillar and fused silica substrate in the mid-infrared are 3.84 and 1.38, respectively. The large refractive index of silicon nanopillar compared to its environment (air, water or oil) makes it possible to provide large phase manipulation and thus regulate light effectively. The phase shifts were calculated by finite-difference time-domain (FDTD) method using a commercial simulation software EastWave. The simulated phase shifts (folded between 0 and 2π) as functions of the nanopillar diameters at wavelengths of 4.0 μm, 4.2 μm, 4.4 μm, 4.6 μm and 4.8 μm are shown with different colors in Figure 2b, named as database in this work, clearly showing that both multiple 2π phase coverage and anomalous dispersions are achieved at these wavelengths, which provide more choices to find adequate silicon nanopillars in database to meet the phase requirements at different wavelengths, thus are crucial to the realization of broadband achromatic metalens. Here, anomalous dispersions mean that unfolded phase shifts do not always decrease or increase monotonically with nanopillar diameters, while phase shifts at some diameters experience a contrary variation tendency to most others.

For normal incident light, the required phase profile of a metalens to achieve diffraction-limited focusing is a function of wavelength:

(1) $$\varphi (\lambda )=\frac{2\pi }{\lambda }(f-\sqrt{r^{2}+f^{2}})$$

where φ(λ) is the phase profile, λ is the wavelength of incident light, r is the distance from arbitrary unit cell on the metalens to the center of the metalens, and f is the focal length [8]. As Equation (1) shows, the required phase profile varies with wavelength. If we design the sizes and spatial distributions of silicon nanopillars according to the phase profile at a certain wavelength, it may not satisfy the required phase profiles at other wavelengths. Moreover, it is difficult to find an adequate spatial distribution of silicon nanopillars with various cross-section sizes that simultaneously satisfies the required phase profiles at all wavelengths in the operating band.

**Figure 3:**
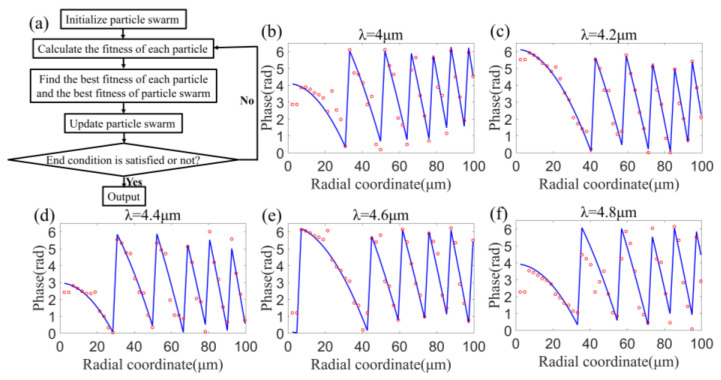
(**a**) Flowchart of PSO algorithm. (**b**–**f**) Comparisons between the corrected required phase profiles (blue line) and the phase profiles induced by the designed metalens (red point) at wavelengths of 4.0 μm (**b**), 4.2 μm (**c**), 4.4 μm (**d**), 4.6 μm (**e**) and 4.8 μm (**f**), as functions of the radial coordinate of the metalens.

Therefore, we introduced a phase correction to the required phase profile:

(2) $$\varphi (\lambda )=\frac{2\pi }{\lambda }(f-\sqrt{r^{2}+f^{2}})+C(\lambda )$$

where φ(λ) is the corrected required phase profile, and C(λ) is the phase correction, which is a function of wavelength [16]. The role of C(λ) is to significantly reduce the difficulty of finding an adequate spatial distribution of silicon nanopillars with various sizes that simultaneously satisfies the corrected required phase profiles at all wavelengths in the operating band. First, we optimized the phase correction by multi-objective optimization methodology based on particle swarm optimization (PSO) algorithm. The flowchart of PSO algorithm is shown in Figure 3a. Then, we designed the sizes and spatial distributions of silicon nanopillars according to the corrected required phase profile and the database mentioned above. After optimization, the total phase difference (referred to $\Delta \varphi_{total}$ in Equation (3)) at all positions of the metalens and all wavelengths between the corrected required phase profile (referred to $\varphi_{required}$ in Equation (3)) and the phase profile induced by the designed metalens (referred to $\varphi_{metalens}$ in Equation (3)) is minimized. The total phase difference is calculated as follows:

(3) $$\Delta \varphi_{total}=\sum_{i}\sum_{n}\left|\varphi_{required}(r_{i},\lambda_{n})\right.-\left.\varphi_{metalens}(r_{i},\lambda_{n})\right|$$

where subscript *i* represents different positions of the designed metalens, and subscript *n* represents different wavelengths in the operating band.

To verify our method, we designed a broadband achromatic metalens over a continuous range of wavelengths from 4.0 to 4.8 μm. The operating band is discretized into five equally spaced wavelengths. The metalens has a diameter of 200 μm, a focal length of 200 μm, giving a numerical aperture of 0.45. The optimized phase correction corresponding to each wavelength is listed in Table 1.

It is worth noting that, the average phase difference at each position of the metalens and at each wavelength, i.e., the total phase difference divided by the number of all positions and all wavelengths, is merely 0.06 * 2π, which indicates nearly perfect focusing and imaging according to the well-known Rayleigh criterion. Figure 3b–f show the comparisons between the corrected required phase profiles (blue line) and the phase profiles induced by the designed metalens (red point) at different wavelengths, as functions of the radial coordinate of the metalens. The phase profiles induced by the designed metalens are consistent with the corrected required phase profiles.

## 3. Results

**Figure 4:**
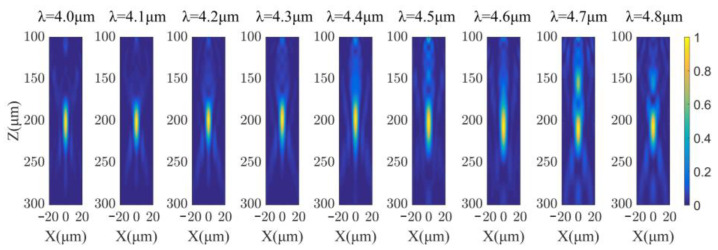
Simulated normalized intensity profiles of the normally incident light transmitted through the metalens in the xz-plane at wavelengths from 4.0 μm to 4.8 μm (spaced by 0.1 μm), where *z*-axis is the optical axis and *x*-axis is the radial direction of the metalens.

We analyzed the optical response of the achromatic metalens by FDTD method using the above-mentioned commercial simulation software EastWave. Figure 4 shows the simulated normalized intensity profiles of the normally incident light transmitted through the metalens in the xz-plane at wavelengths from 4.0 to 4.8 μm (spaced by 0.1 μm), where z-axis is the optical axis and *x*-axis is the radial direction of the metalens. According to Figure 4, the focal length (shown in Table 2) over the whole operating band is nearly constant, which is consistent with the designed focal length (200 μm), with standard deviations of 4 μm, or 2% of focal length. Therefore, the effectiveness of the above optimization method and the broadband achromatism of the designed metalens are verified.

**Figure 5:**
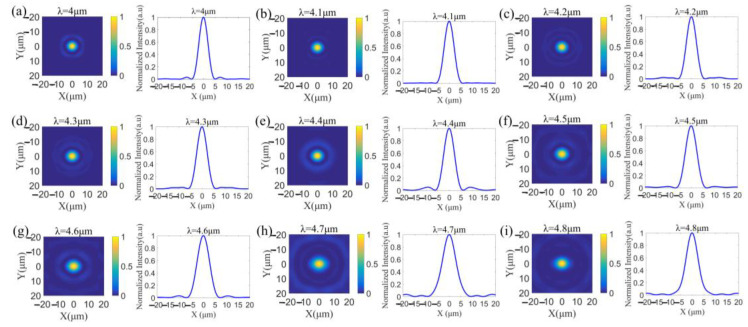
Simulated normalized intensity profiles in the xy-plane (left panels of (**a**–**i**)) at the fixed position of z = 200 μm, i.e., the designed focal plane, at wavelengths from 4.0 μm to 4.8 μm (spaced by 0.1 μm) (**a**–**i**), showing strong focal spots with weak sidelobes. Simulated normalized light intensity along the x-axis (right panels of (**a**–**i**)) at the focal plane, i.e., the horizontal cuts of the focal spots.

To further verify the performance of the achromatic metalens, we simulated the normalized intensity profiles in the xy-plane at the fixed position of z = 200 μm, i.e., the designed focal plane, at wavelengths from 4.0 to 4.8 μm (spaced by 0.1 μm). The strong focal spots with weak sidelobes over the whole operating band are clearly shown in Figure 5. According to the normalized light intensity along the *x*-axis, i.e., the horizontal cuts of the focal spots at z = 200 μm plane, the full widths at half-maximum (FWHMs) at wavelengths from 4.0 to 4.8 μm are calculated and listed in Table 3, revealing near-diffraction-limited focusing performance of the broadband achromatic metalens.

**Figure 6:**
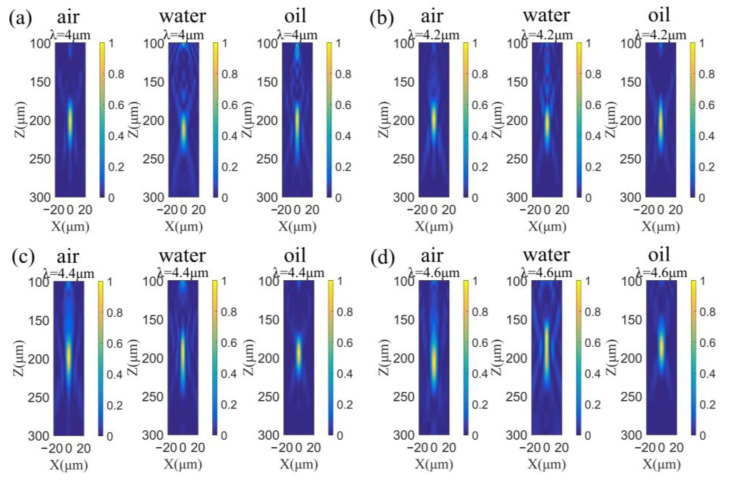
Focusing performance of the designed metalens at wavelengths of 4.0 μm (**a**), 4.2 μm (**b**), 4.4 μm (**c**) and 4.6 μm (**d**), when the metalens is under the environment of water or oil.

To verify the environmental adaptability of the designed metalens, we changed the background material from air to water and oil, corresponding to the case of outdoors equipment exposed to rain and equipment covered by electric insulating oil, respectively. Figure 6 shows the simulated normalized intensity profiles of the normally incident light transmitted through the metalens in the xz-plane at wavelengths of 4.0 μm (a), 4.2 μm (b), 4.4 μm (c) and 4.6 μm (d), where z-axis is the optical axis and x-axis is the radial direction of the metalens. As shown in Figure 6, when the metalens is under the environment of water or oil, the focusing performance of the designed metalens maintains well overall, with slight changes in focal lengths, but the depths of foci still cover the designed focal plane z = 200 μm. These results numerically verify the environmental adaptability of the designed metalens. Compared to the previous reports of achromatic metalens [16,17,18,19,20,21,22,23,24,25,30,31,32,33], which only worked in the air environment, the notable point of this work is providing the feasibility of constructing an all-dielectric mid-infrared metalens with appreciable focusing performance when it is surrounded with air, water and oil, thus further facilitates the practical application of metalens.

To practically obtain the proposed metalens, the feasible fabrication steps are as follows. First, amorphous silicon is deposited on the fused silica substrate by chemical vapor deposition (CVD). Second, PMMA4 photoresist is spin-coated on the wafer. Third, patterns of the metalens are developed on the wafer by electron beam lithography (EBL) and the following steps of developing and removing the exposed photoresist. Fourth, hard mask is developed on the pattern by depositing a layer of chromium by electron beam evaporation and removing the remaining photoresist. Fifth, etching of the metalens is performed by inductively coupled plasma (ICP). Sixth, the hard mask is removed. After these steps, the metalens is fabricated and can be used in practical application. The flowchart of the fabricating method is shown in Figure S1.

## 4. Conclusions

In this work, we numerically demonstrated a broadband achromatic infrared all-dielectric metalens over a continuous 800 nm bandwidth, with environmental adaptability in air, water and oil. By building a database with multiple 2π phase coverage and anomalous dispersions, optimizing the corrected required phase profiles using particle swarm optimization algorithm, and designing the sizes and spatial distributions of silicon nanopillars according to the corrected required phase profile and the database, we numerically realized the design and optimization of a broadband achromatic all-dielectric metalens. The simulation results of the designed metalens show nearly constant focal lengths with standard deviations of merely 2% of focal length and diffraction-limited focal spots over the continuous range of wavelengths from 4.0 μm to 4.8 μm, revealing the achromatic response of the metalens. The broad bandwidth indicates the ability of the designed metalens to detect thermal signals over a temperature range from various fault points. Further simulation results show that the metalens maintains good focusing performance under the environment of water or oil. This work may facilitate the application of metalens in ultra-compact infrared detectors for power grid faults detection.

## Figures and Tables

**Table 1:** Optimized phase correction at wavelengths from 4.0 to 4.8 μm.

Wavelength (μm)	4.0	4.2	4.4	4.6	4.8
C(λ) (rad)	−2.174	−0.141	−15.872	0.0949	29.065

**Table 2:** Focal lengths at wavelengths from 4.0 to 4.8 μm.

Wavelength (μm)	4.0	4.1	4.2	4.3	4.4	4.5	4.6	4.7	4.8
Focal length (μm)	202	202	200	198	198	202	207	210	207

**Table 3:** FWHMs at wavelengths from 4.0 to 4.8 μm.

Wavelength (μm)	4.0	4.1	4.2	4.3	4.4	4.5	4.6	4.7	4.8
FWHM (μm)	4.6	5.0	5.0	5.0	5.4	5.6	6.2	6.6	5.8

## Data Availability

The data presented in this study are available on request from the corresponding author. The data are not publicly available due to the authors haven’t uploaded it to publicly accessible repository.

## Supplementary Materials

The following supporting information can be downloaded at: https://www.mdpi.com/article/10.3390/s22176590/s1, Figure S1. The flowchart of fabricating method. Figure S2. The set-up diagram of measuring method.
