# Supplementary material for: Robust Achromatic All-Dielectric Metalens for Infrared Detection in Intelligent Inspection
Source: Sensors (Basel). 2022 Aug 31;22(17):6590. doi: 10.3390/s22176590 (PMC9460807; doi:10.3390/s22176590)
Supplement: Supplementary file 1 [file sensors-22-06590-s001.zip › sensors-1857124-supplementary.pdf]

Supplementary material

Robust achromatic all-dielectric metalens for infrared detection in intelligent inspection

Wenrong Si, Zhengyong Hu, Dun Lan, Yi Zhou and Wei Li

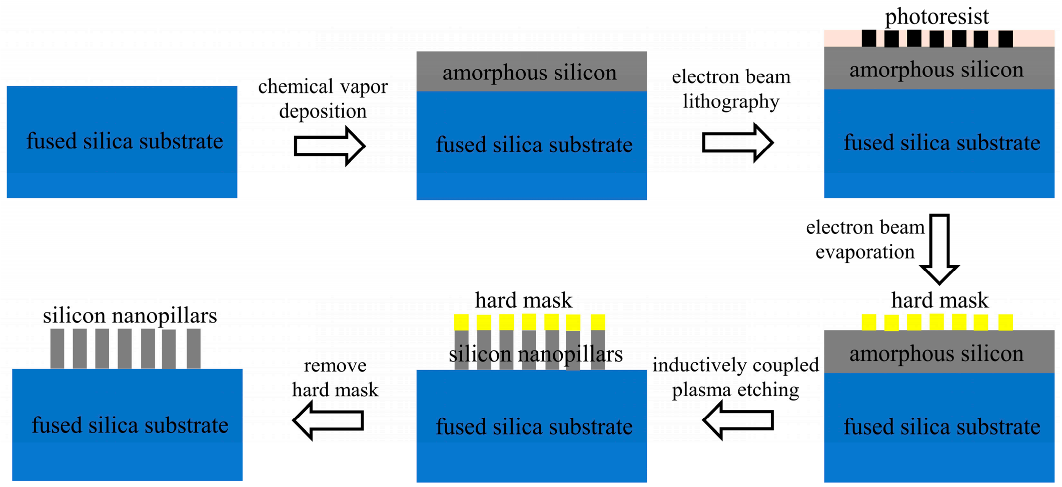

Figure S1. The flowchart of fabricating method.

The experimental setup to validate the robust focusing performance of the designed achromatic metalens is shown in Figure S2. The incident light source first propagates through the optical bandpass filter to select the incident wavelength, next propagates through the polarizer to be polarized as a linear-polarized light. Then, the linear-polarized light is focused by the metalens sample. Finally, an objective lens and a tube lens are used to image the focus on the camera. To locate the accurate focal plane, a motorized translation stage (black dashed box in Figure S2) is moved along the z-direction (i.e. propagating direction) in steps of 2μm to capture images in each xy-plane at different positions of z. To validate the environmental adaptability of the metalens, metalens sample is encapsulated in a transparent box (black solid box in Figure S2). By injecting water or oil into the box, the surrounding environment of metalens sample is changed and the images of focus are collected at each case.

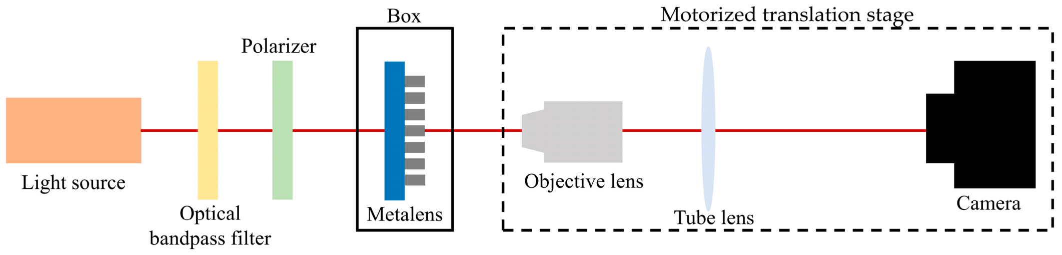

Figure S2. The set-up diagram of measuring method.
